# The management of patients with metastatic prostate cancer during the COVID-19 pandemic

**DOI:** 10.2217/fon-2020-0361

**Published:** 2020-05-15

**Authors:** Tarek Assi, Nathalie Ibrahim, Rita-Maria K Abboud, Clarisse Kattan, Elie Rassy, Elie Nemr, Joseph Kattan

**Affiliations:** 1 Department of Hematology-Oncology, Faculty of Medicine, Saint-Joseph University, Beirut, BP 17-5208, Lebanon; 2 Department of Urology, Faculty of Medicine, Saint-Joseph University, Beirut, BP 17-5208, Lebanon

**Keywords:** castrate resistant, coronavirus, COVID-19, hormone sensitive, immunosuppression, metastatic, prostate cancer

## Abstract

During the ongoing global pandemic of coronavirus disease 2019 (COVID-19), the benefit of treating patients with cancer must be weighed against the COVID-19 infection risks to patients, staff and society. Prostate cancer is one of the most common cancers among men and raises particular interest during the pandemic as recent reports show that the TMPRSS2 (and other serine proteases), which facilitate the entry, replication and budding of the virion from a cell, can be inhibited using androgen deprivation therapy. Nevertheless, patients with metastatic prostate cancer commonly receive chemotherapy which may compromise their immune system. This paper aims to address the current status of the COVID-19 in patients with cancer overall and suggests an optimal approach to patients with metastatic prostate cancer.

With the surge of the SARS coronavirus-2 (also known as COVID-19) pandemic worldwide, the oncology community is heavily investing the possible resources to optimize the management of patients with cancer [1]. By the 30th April 2020, more than 300,000 people had confirmed COVID-19 cases and more than 215,000 patients have already succumbed to the infection [2]. This pathogen has raised concerns with severe complications, notably among cancer patients [3,4]. Multiple recommendations have been published to help the medical community in optimizing the therapeutic strategies of cancer patients but the current literature still lacks supporting evidence [5–9]. Given the paucity of evidence-based decisions at this point of the COVID-19 pandemic, most measures adopted have been based on data extrapolated from other coronavirus infections and are influenced by expert opinion. The roadmap to manage cancer patients opts to minimize the number of hospital visits and hospitalizations and to prevent anticancer treatment–induced complications of COVID-19 [10]. Prostate cancer ranks among the most frequent tumors and is a topic of particular interest during the pandemic as TMPRSS2 (and other serine proteases), which facilitate the entry, replication and budding of the virion from a cell can be inhibited using androgen deprivation therapy (ADT) [11–13]. In the metastatic setting, chemotherapy known to improve the survival of patients may compromise the immune system of patients; hence, the need for an adapted sequencing of the treatment regimens to minimize the risk of COVID-19 infection. This paper aims to address the current status of the COVID-19 in patients with cancer overall and suggests an optimal approach to patients with metastatic prostate cancer.

## COVID-19 & cancer

The Chinese centers have shared their experience on the COVID-19-related outcomes in cancer patients since the beginning of the COVID-19 outbreak [14]. A nationwide Chinese cohort of 1590 COVID-19 patients reported a higher percentage of cancer patients in the COVID-19 cohort than in the overall population (1 vs 0.29%) [15]. It included 18 cancer patients among which 12 patients were long-term cancer survivors with no active cancer. The risk of severe events among these patients was higher than those without cancer (39 vs 8%; p = 0.0003) and correlated with a history of chemotherapy or surgery in the month preceding infection (odds ratio: 5.40; 95% CI: 1.80–16.18). Patients with cancer deteriorated more rapidly than those without cancer (13 vs 43 days; p < 0.001; hazard ratio [HR]: 3.56; 95% CI: 1.65–7.69) and had an overall case fatality rate of 5.6% [15]. A case series of 67 cancer patients infected with COVID-19 have identified 23 patients (34.3%) who were on active anticancer therapy. The encountered cases were predominantly patients with lung cancers (22.4%) followed by colorectal, thyroid and urinary tumors in 16.4, 11.9 and 11.9%, respectively. Cancer patients were reported to have a higher risk of severe COVID-19 infections in comparison with the general population (47.7 vs 15.6%) [16,17]. Compared with the mild illness group, patients in the severe illness group were older (69 vs 64 years; p < 0.001) and had more comorbidities (71.9 vs 37.1%; p = 0.004). Patients at the follow-up phase seem to have a better prognosis than patients in the active treatment phase (case fatality rate: 20.5 vs 39.1%; p = 0.095) [16]. Another case series of 28 cancer patients reported severe events in 54% of cases, notably among patients receiving antitumor treatment within 14 days (odds ratio: 4.079; 95% CI: 1.086–15.322). Lung cancer (25%), esophageal cancer (14%) and breast cancer (11%) were the most commonly encountered tumors. Empirical antibiotics, antiviral agents and intravenous immunoglobulins were administered in 82, 71 and 26%, respectively. The mortality rate reached 29% and invasive mechanical intubation was required in 36% of cases [18].

## Metastatic prostate cancer

Prostate cancer is the most common urological cancer and the second cause of cancer deaths among men [19]. Medical castration represents the main treatment approach in patients with metastatic hormone-sensitive prostate cancer (mHSPC) but required additional anticancer treatments as it becomes metastatic castrate-resistant prostate cancer (mCRPC) [20]. In the last two decades, the treatment landscape of mCRPC has been transformed with the addition of chemotherapeutic agents (docetaxel and cabazitaxel) and hormonal agents (abiraterone acetate, enzalutamide, apalutamide and darolutamide) [21–23]. This success in the mCRPC setting encouraged researchers to evaluate the role of these agents at an earlier setting in the hormone-sensitive setting which has largely changed the treatment sequencing of prostate cancer patients [24,25]. With the novel outbreak of the COVID-19 infection, special considerations should be undertaken in the treatment selection of metastatic prostate cancer patients, more particularly due to the advanced age and frailty of these patients at diagnosis.

## Metastatic hormone-sensitive prostate cancer

In the mHSPC setting, three antihormonal agents (apalutamide, enzalutamide and abiraterone acetate) and docetaxel were approved in addition to ADT. The role of docetaxel in the mHSPC was assessed in three large randomized trials [26–28]. The French trial, GETUG-AFU 15, randomized 285 patients to ADT alone or in combination with docetaxel (75 mg/sqm every 3 weeks up to nine cycles). The median overall survival (OS) showed similar outcomes between the two treatment arms (58.9 vs 54.2 months in the experimental and control arm, respectively [HR: 1.01; 95% CI: 0.75–1.36]) [27]. The American trial, CHAARTED, randomized 790 patients to ADT alone or in combination to docetaxel (75 mg/sqm every 3 weeks up to six cycles), which yielded higher median OS in the combination arm (HR: 0.61; 95% CI: 0.47–0.80; p < 0.001) with higher benefit in patients with high-volume disease (51.2 vs 34.4 months in favor of combination arm; HR: 0.63; 95% CI: 0.50–0.79; p < 0.001) [24,28]. At last, the English trial, STAMPEDE, had a similar design to the CHAARTED trial except for prednisolone 10 mg daily that was added to docetaxel. It revealed a significantly higher median OS benefit in the combination arm (71 vs 81 months; HR: 0.78; 95% CI: 0.66–0.93; p = 0.006) [26]. Subsequently, docetaxel was approved in the specific category of high-risk mCRPC [26,28]. It is noteworthy that grade 3–4 neutropenia was reported in 12–32% of patients and febrile neutropenia in 7–16% of patients [29]. Another analysis from the STAMPEDE trial included 2061 patients with mHSPC that were randomized to receive standard therapy with or without radiotherapy to the primary tumor (3–4 weeks after the last docetaxel dose). Overall, radiation therapy did not improve the survival in newly diagnosed patients with mHSPC but those with limited tumor burden had significant improvement in 3-year OS (81 vs 73%; HR: 0.68; 95% CI: 0.52–0.9; p = 0.0007) [30].

Hormonal agents were also shown effective and less toxic in patients with mHSPC. The LATITUDE trial randomized 1199 patients to receive ADT alone or in combination with abiraterone acetate. The combination regimen had a longer median OS in comparison with ADT monotherapy (53.3 vs 36.6 months; HR: 0.66; 95% CI: 0.56–0.78; p < 0.0001) [25,31]. Grade 3–5 neutropenia was reported in less than 2% in both arms with no reported cases of febrile neutropenia [31]. The ENZAMET trial randomized 1125 patients to ADT monotherapy or in combination with enzalutamide. The 3-year OS favored the combination arm (80 vs 72%; HR: 0.67; 95% CI: 0.52–0.86; p = 0.002) [32]. Grade 3 or more neutropenia was reported in 6% and febrile neutropenia in 7% [32]. The TITAN trial randomized 525 patients to ADT alone or in combination with apalutamide. The combination arm yielded higher 2-year OS (82.4 vs 73.5%; HR: 0.67; 95% CI: 0.51–0.89; p = 0.005) [22]. Grade 3 or more neutropenia and febrile neutropenia episodes were only reported in 0.4 and 0.2% of patients, respectively [22].

As a result, novel hormonal therapy in combination with ADT seems a more plausible option among patients with newly diagnosed mHSPC as the risk of neutropenia is lower (less than 6% vs more than 30% with chemotherapeutic agents). Published data reporting on the safety of ADT during the COVID-19 pandemic are rare with one small retrospective series suggesting a lower prevalence of COVID-19 infection among patients undergoing ADT and/or 5-alpha reductase inhibitors compared with men older than 40 years (4.2 vs 14.9%; p < 0.0001) [33]. Docetaxel can be safely postponed until later lines after the control of the pandemic in a subset of patients with low tumor burden. Case-by-case discussion should be performed in patients with aggressive visceral involvement and highly symptomatic disease. The choice of therapy is also backed up by the results of a network meta-analysis that evaluated the currently approved options in the mHSPC setting and favored the use of abiraterone acetate over docetaxel-based therapy [29]. The STAMPEDE trial also compared abiraterone acetate and docetaxel in patients with mHSPC and showed no evidence of a difference in overall or prostate cancer-specific survival, or other important outcomes such as symptomatic skeletal events. The worst toxicity grade was similar but comprised different toxicities in line with the known properties of the drugs [34].

Hypertension is a common side effect of the novel antihormonal therapy (abiraterone acetate and enzalutamide) and is mainly managed using ACE inhibitors [31,32]. It has been demonstrated that the COVID-19 binds to the target cells through ACE2 which is expressed on the epithelial cells of different organs [35]. ACE2 is upregulated in patients treated with ACE inhibitors thus it is thought that patients with hypertension receiving antihormonal agents might be at higher risk of infection with the COVID-19 [36]. These findings should be taken into consideration when a choice of therapy between oral and intravenous agents must be made.

## Metastatic castrate-resistant prostate cancer

In the mCRPC setting, two chemotherapeutic agents are widely used including docetaxel as the initial choice followed by cabazitaxel in patients previously treated with docetaxel. Docetaxel was the first active cytotoxic agent to be approved in 2004 for mCRPC patients based on the outcomes of two landmark Phase III trials showing significant survival benefit [37,38]. The SWOG 9916 trial is a Phase III study of 770 mCRPC patients that were randomized to docetaxel (60 mg/sqm plus dexamethasone every 3 weeks) and estramustine (280 mg three-times daily from day 1 till 5) or mitoxantrone plus prednisone. The docetaxel arm achieved better OS (HR: 0.80; 95% CI: 0.67–0.97; p = 0.02) and biological response (50 vs 27%, p < 0.001). Grade 3 or more neutropenia in patients treated with docetaxel occurred in 16.5% while febrile neutropenia was reported in 5% of cases [38]. The TAX 327 trial is a Phase III study of 1006 mCRPC patients that were randomized to docetaxel with prednisone (5 mg twice daily) or mitoxantrone with prednisone. In comparison with mitoxantrone, docetaxel every 3 weeks yielded better median OS (18.9 vs 16.5 months; HR: 0.76; 95% CI: 0.62–0.94; p = 0.009), biological response (45 vs 32%, p < 0.001) and symptomatic relief (35 vs 22% for pain reduction, p = 0.01) [37,39]. Grade 3 or more neutropenia was reported in 32% of patients with 3% of febrile neutropenia in the docetaxel arm. The TROPIC trial randomized 755 mCRPC patients progressing after docetaxel to receive cabazitaxel (25 mg/sqm every 3 weeks) or mitoxantrone and showed that cabazitaxel yielded a longer median OS (15.1 vs 12.7 months; HR: 0.7; 95% CI: 0.59–0.83; p < 0.0001). Grade 3–5 neutropenia was reported in 82% of patients and febrile neutropenia in 8% [40]. In the noninferiority PROSELICA trial, 1200 mCRPC patients were randomized to cabazitaxel postdocetaxel at 20 or 25 mg/sqm. The two treatment arms showed similar outcomes but cabazitaxel 20 mg/sqm showed lower rates of grade 3 or more neutropenia (41.8 vs 73.3%) and febrile neutropenia (2.1 vs 9.2%) [41].

The use of the novel hormonal agents in the mCRPC setting has demonstrated encouraging results in terms of survival with minimal toxicity. In the COU-301 trial, abiraterone acetate after progression to docetaxel improved the median OS in comparison with placebo (15.8 vs 11.2 months; HR: 0.74; 95% CI: 0.64–0.86; p < 0.0001). Similarly, the COU-302 showed that abiraterone acetate also improved OS in chemotherapy-naive patients in comparison with placebo (34.7 vs 30.3 months; HR: 0.81; 95% CI: 0.70–0.93; p = 0.0033). Severe neutropenia and febrile episodes were reported in less than 1% of cases [42,43]. Enzalutamide was also assessed in two different trials among patients with mCRPC. The AFFIRM trial tested enzalutamide after failure of docetaxel and the PREVAIL in the chemotherapy-naive setting. In both trials, the median OS was significantly improved in favor of enzalutamide: median OS 8.3 versus 2.9 months (HR: 0.4; 95% CI: 0.35–0.47; p < 0.001) in the former trial and 18.4 versus 13.6 months (HR: 0.63; 95% CI: 0.53–0.75; p < 0.001) in the latter study. No neutropenic events were reported [44,45].

More recently, the CARD trial randomized 225 patients with mCRPC between cabazitaxel or the other antihormonal agents (enzalutamide or abiraterone acetate). Cabazitaxel had improved the median OS in comparison with the other arm (13.6 vs 11 months; HR: 0.64; 95% CI: 0.46–0.89; p = 0.008) [46]. More grade 3–5 neutropenia (44.7 vs 3.2%) and febrile neutropenia (3.2 vs 0%) were reported with cabazitaxel in comparison with antihormonal agents [46].

## Conclusion & Future perspective

The chemotherapeutic agents, docetaxel and cabazitaxel, are associated with a substantial risk of neutropenia that could predispose patients to the risk of the COVID-19 infection. These regimens commonly require hospital admission in the same-day unit departments for intravenous perfusions which expose the patients and the personnel to the risk of infection [18]. Some practices have suggested home care to start giving chemotherapy at home under strict surveillance, but have not been consistently adopted by medical oncology societies. Antihormonal therapy including ADT and other novel antihormonal therapies are associated with a lower prevalence of neutropenia compared with chemotherapy. Thus, the novel hormonal therapy may be a safer option with a slight privilege for enzalutamide which does not require prednisone supplementation, more particularly in endemic areas where access to home nursing and at-home chemotherapy administrations is limited.

Patients diagnosed with mHNPC can be safely treated with ADT plus novel hormonal therapy. Besides, the radiotherapy of the primary tumor in patients with low tumor burden can be postponed. Patients with mCRPC can be managed by peripheral ADT withdrawal or the addition of bicalutamide as it may delay, though for a short period, the administration of chemotherapeutic agents [47,48]. In the particular case of mCRPC that have previously received docetaxel and one of the novel antihormonal therapies, cabazitaxel remains the optimal agent [40,41]. However, in an effort to delay chemotherapy, patients with slow growing mCRPC could be managed exceptionally with sequential novel antihormonal therapies, even if there is a known cross-resistance between these agents. Otherwise cabazitaxel can be administered at 20 mg/sqm every 3 weeks followed by granulocyte colony stimulating factors. Small doses of corticosteroids are used in combination with active anticancer agents, but strict measures should be undertaken to prevent the occurrence of infections with the long-term effect of steroids and their immunosuppressive characteristics [49]. The use of bisphosphonates as bone protective agents can be minimized to biyearly administration or replaced by subcutaneous denosumab in order to reduce hospitalization for intravenous perfusions and the risk of complications, mainly osteonecrosis of the jaw [43,50]. Concerning clinical trials, the US FDA and the EMA have issued special guidance for the conduction of clinical trials during the COVID-19 pandemic [51,52].

Medical oncologists are sharing their efforts and joint experience to preserve the continuity of cancer care amidst the challenges imposed by the current COVID-19 pandemic. The ongoing research to identify an antiviral therapy and to close key knowledge gaps about the incidence, morbidity and mortality of COVID-19 specific to cancer patients [53,54]. We have provided practical guidance for prostate cancer treatment that the oncologist should take into consideration to personalize treatment decisions based case-by-case discussion.

**Executive summary**

**Background**

- COVID-19 may be associated with higher complications in cancer patients.
- Multiple recommendations have been issued to optimize the therapeutic strategies of cancer patients but the current literature still lacks guidance for prostate cancer patients.
- The risk of severe events among cancer patients was higher than those without cancer (39 vs 8%) and correlated with a history of chemotherapy or surgery in the month preceding infection.
- Cancer patients deteriorated more rapidly and had an overall case fatality rate of 5.6%.

**Metastatic hormone-sensitive prostate cancer**

- In the metastatic hormone-sensitive prostate cancer setting, three antihormonal agents (apalutamide, enzalutamide and abiraterone acetate) and docetaxel were approved in addition to androgen deprivation therapy (ADT).
- Docetaxel significantly improved the overall survival (OS) outcomes and was associated with grade 3–4 neutropenia in 12–32%, and febrile neutropenia in 7–16%.
- Antihormonal agents (apalutamide, enzalutamide and abiraterone acetate) were also shown effective with significant impact on OS and less toxicity (grade 3–4 neutropenia in 0.4–6% and febrile neutropenia in 0.2–7%).

**Metastatic castrate-resistant prostate cancer**

- In the metastatic castrate-resistant prostate cancer (mCRPC) setting, docetaxel yielded a significant OS benefit and was associated with grade 3–4 in 16.5–32% and febrile neutropenia in 3–4%.
- Cabazitaxel in docetaxel-refractory patients demonstrated improved OS but with higher rate of neutropenic events; grade 3–4 neutropenia in 82% of patients and febrile neutropenia in 8%.
- Both abiraterone acetate and enzalutamide improved OS in this setting with less than 1% reported neutropenia or febrile neutropenia cases.
- In the CARD trial, cabazitaxel improved the OS in comparison with hormonal agents but higher neutropenic events were noted.

**Conclusion**

- The chemotherapeutic agents, docetaxel and cabazitaxel, are associated with a substantial risk of neutropenia that could predispose patients to the risk of COVID-19 infection.
- Novel antihormonal therapy in combination with ADT seems a plausible option among patients with newly diagnosed metastatic hormone-sensitive prostate cancer and docetaxel can be safely postponed until later lines in a subset of patients with low tumor burden after the control of the pandemic.
- Radiotherapy of the primary tumor in patients with low tumor burden can be safely postponed.
- Patients with mCRPC can be managed by peripheral ADT withdrawal or the addition of bicalutamide.
- Patients with slow-growing mCRPC could be managed exceptionally with sequential novel antihormonal therapies, even if there is a known cross-resistance between these agents.
